# Genetic profiling of the *Plasmodium falciparum* parasite population in uncomplicated malaria from India

**DOI:** 10.1186/s12936-019-3022-5

**Published:** 2019-12-02

**Authors:** Amit Kumar, Shri Pat Singh, Rajendra Bhatt, Vineeta Singh

**Affiliations:** ICMR-National Institute of Malaria Research, Sector-8, Dwarka, New Delhi, India

**Keywords:** *Plasmodium falciparum*, Drug resistance genotyping, *Pfg377*gene

## Abstract

**Background:**

The genetic complexity and the existence of several polymorphisms in parasites are the major hindrances for the malaria control programmes of the country. The genetic profiling in the parasite populations in India will provide useful baseline data for future studies elucidating the parasite structure and distribution of drug resistance genotypes in different regions.

**Methods:**

The blood samples of symptomatic patients were collected and analysed for drug resistance genes (*Pfcrt*, *Pfmdr*-*1*, *dhfr*, *dhps* and *k13*) and gametocyte genes (*Pfs25*, *Pfg377*); in vitro drug sensitivity assay by schizont maturation inhibition (SMI) was also performed in adapted field isolates.

**Results:**

Of the 122 field isolates analysed; 65.5% showed *Pfcrt* K76T mutant alleles, 61.4% *Pfmdr*-*1* N86Y mutants, 59.5% *dhfr* mutants, 59.8% *dhps* mutants was observed, but no polymorphism was seen for *k13*. The sequence analysis of *Pfg377* gene revealed five types of populations in the field isolates. The inhibitory concentrations (IC_50_) for anti-malarial drugs viz chloroquine (CQ), artesunate (AS), were in the range of 10.11–113.2 nM and 2.26–4.08 nM, respectively, in the field isolates evaluate by in vitro assay. The IC_50_ values for CQ have shown a remarkable reduction on comparison with the previous available data, whereas a slight increase in the IC_50_ values for AS was observed in the study.

**Conclusions:**

The increase in mutation rate in drug resistance allelic loci with inhibitory concentration of CQ and AS drugs was observed in the field isolates and high diversity in *Pfg377* gametocyte gene indicate towards parasite multifactorial behaviour. The knowledge of the prevalent drug resistance genes is important for intervention measures to be successful and efforts should also be made to prevent transmission of *P. falciparum*.

## Background

Morbidity and mortality due to *Plasmodium falciparum* malaria have increased across Indian sub-geographical regions, largely because of the widespread resistance to chloroquine (CQ) and sulfadoxine–pyrimethamine (SP) leading to generation of regional heterogeneity in the target genes [1, 2]. The rise in the incidences of malaria is largely attributed to the increase in the drug resistance parasites [3]. The emergence of chloroquine resistance in India led to the change in the drug policy in the year 2005 to artemisinin-based combination therapy (ACT) as first-line of treatment [4]. The *Pfcrt* and *Pfmdr*-*1* genes are associated with CQ resistance, whereas the *dhfr* and *dhps* genes are related with SP resistance in the malaria parasite caused by the mutations present in these drug resistance genes [5]. The Kelch propeller domain (*k13*-propeller) in *P. falciparum* have been found to be associated with delayed parasite clearance after ACT therapy [6].

*Plasmodium falciparum* is transmitted from the human host to the mosquito vector during the sexual stage via gametocyte. But the transmissibility of *P. falciparum* is due to the relationship between the prevalence, duration and density of gametocyte carriage with variable and modulated by the immune response in the human host [7]. Gametocytes from multi-clone *P. falciparum* infections can persist three times longer than single-clone infections indicating that multiplicity of infection (MOI) promotes either longer persistence or continuous production of gametocytes [8]. The occurrence of sub-microscopic gametocytes in the parasite population play a major role in malaria transmission. It has also been shown that the microscopic diagnosis is more likely to underestimate the prevalence of gametocytes and more so in the sub-patent population of gametocytes [9]. For the successful elimination of *P. falciparum* it is crucial to limit the transmission of malaria parasites which can be achieved by reducing the carriage of viable gametocytes, hence it is important to investigate the drug resistance genes with gametocyte genes [9].

The aims of the present study were to determine the prevalence of *Pfcrt*, *Pfmdr*-*1*, *dhfr* and *dhps* mutations in drug resistance genes with *k13* (particularly ACT with SP as partner drug and CQ) and *Pfg377* genes with in vitro susceptibility assays in order to understand the genetic complexity of falciparum malaria in natural infections. The *Pfcrt*, *Pfmdr*-*1*, *dhfr* and *dhps* are directly implicated in parasite population turning resistant to anti-malarials and *Pfg377* gene is considered under neutral/balancing selection. Coupling the analysis of drug resistance genes along with *k13* with in vitro studies will give an insight about the circulating parasite population in the region and profiling the chloroquine markers will provide data to know if there is any change at the genetic level for these markers after a change in the treatment policy.

## Methods

### Study population and ethics

**Fig. 1:**
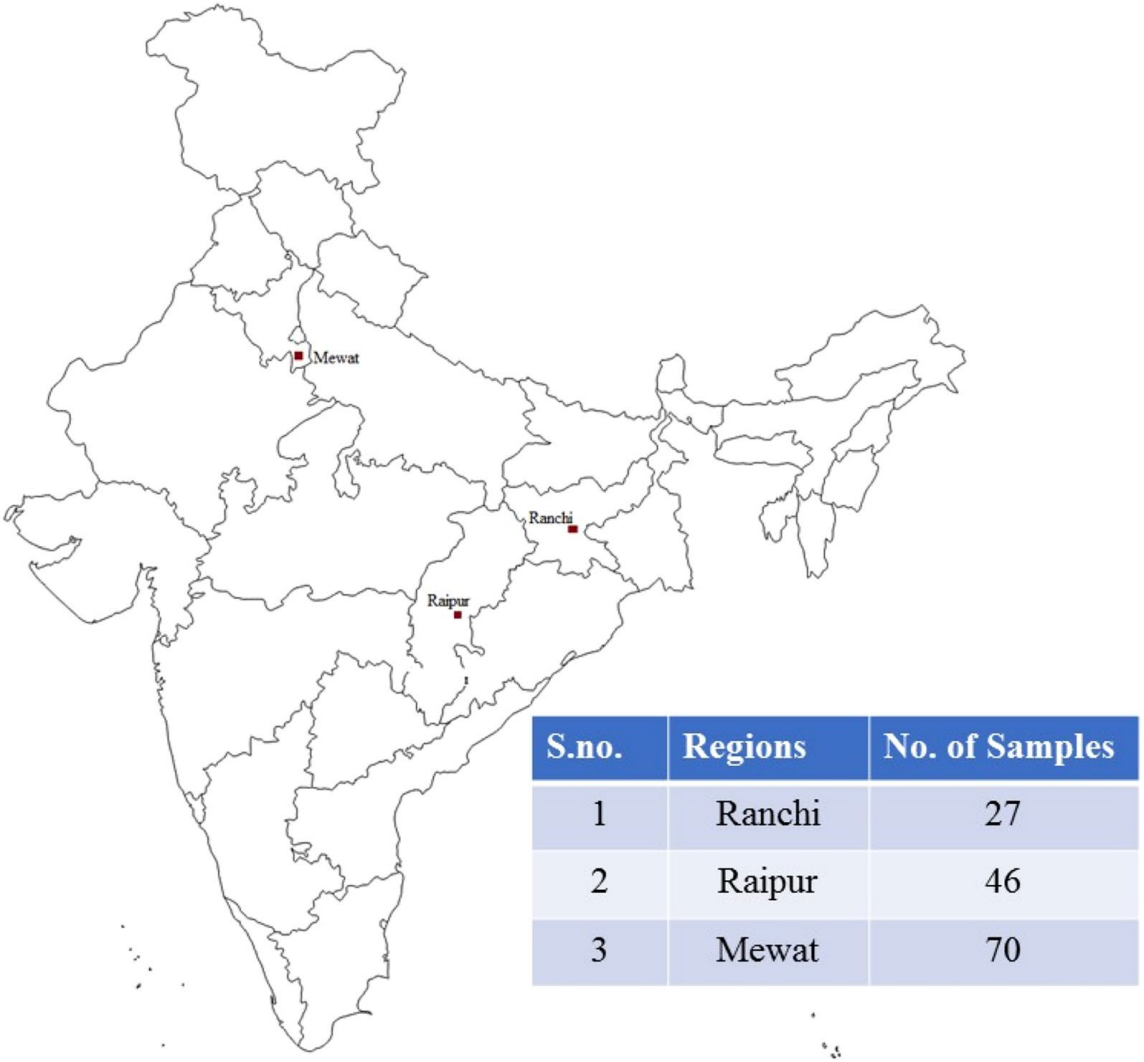
Geographic map of India showing the study sites viz Mewat, Raipur and Ranchi with number of *P. falciparum* samples collected from the these sites

In the present study, 143 malaria blood samples were collected from the primary health centres (PHCs) in Mewat, Raipur and Rourkela during the transmission season in the years 2015–2016 (Fig. 1). The study group comprised of patients of either sex, 5–60 years of age, with symptoms of malaria, but no history of prior medication until previous 30 days. Two microliter of venous blood from patients found to be *P. falciparum* positive by microscopy/RDT were collected in EDTA vacutainer tubes. 100 µl of the collected sample was used for filter paper spots, labelled and transported in the laboratory in sealed plastic envelopes and remaining samples were cryopreserved.

The institutional ethics committee (NIMR-IEC) approved the study (reference no. ECR/NIMR/EC/2) and the ethical guidelines of the Institute were followed for sample collection. Verbal and written informed consent was obtained from all patients/guardians in case of minors.

### Nucleic acid extraction

The parasite genomic DNA from filter paper was extracted using QIAamp TM DNA mini kit (QIAGEN), according to the manufacturer’s instructions (dried blood spots protocol) with slight modifications. DNA samples were kept at − 20 °C until further processing.

### PCR analysis

PCR assay for *Plasmodium* species: After the DNA extraction nested PCR assay with 18S rRNA primers was performed to differentiate between the mixed infections of *Plasmodium vivax* and *P. falciparum*, as described previously [10].

### Genotyping of drug resistance genes

The *Pfcrt* gene codon-76; *Pfmdr*-*1* codon-86, *dhfr* codons-16, 51, 59, 108, 164; *dhps* gene codons-436, 437, 540, 581, 613 and *k13* gene were analysed as described earlier [6, 11, 12]. All PCR amplifications were performed with 1 µl of template DNA from samples including the negative controls and reference strain 3D7 as standard positive controls.

### PCR–RFLP assay

After successful PCR amplification the polymorphisms in *Pfcrt* and *Pfmdr*-*1* genes were determined by RFLP assay by digestion with ApoI and AflIII enzymes. The enzyme ApoI cleaves the *Pfcrt*-76K (the CQ sensitive isolates) but not *Pfcrt*-76T (as seen in CQ resistant isolates) and allele of *Pfmdr*-*1*-86N (the CQ sensitive isolates) but not *Pfmdr*-*1*-86Y (the CQ resistant isolates). AflIII was unable to digest the amplicon of the sensitive isolates.

### Sequence analysis

The *k13* amplification reactions were carried out as described earlier and mutations were assessed by comparing each sequence with the reference 3D7 K13-propeller (PF3D7_1343700) [6]. The positive PCR products of *dhfr* and *dhps* genes were purified and sequenced on an ABI 3730 DNA analyzer. The 3D7 strain (GenBank ID: for *dhfr* 9221804 and *dhps* 2655294) was used as reference for comparing the gene sequences. The sequence comparisons was done with the Plasmo database of these two genes with basic local alignment search too N (BALST N) and MEGA software [13].

### Culture studies

#### Parasite culture

In the laboratory, the cryopreserved samples were revived in the RPMI 1640 culture medium supplemented with gentamicin (0.01 mg/ml), 25 mM Hepes buffer, 25 mM NaHCO_3_ and 5% human serum maintained in 5% CO_2_ with incubation at 37 °C. The growth of the parasite in vitro was visualized daily by microscopic examination of the Giemsa-stained blood films [14].

#### Gametocyte production

The field isolates of *P. falciparum* were put to gametocyte production at 0.5% parasitaemia and 6% haematocrit under 3% O_2_, 5% CO_2_, 92% N_2_ gas. RPMI culture medium with 25 mM HEPES, 50 mg/l hypoxanthine, 2 g/litre sodium bicarbonate, 10% human serum, no fresh erythrocytes were added to culture till 14 days. Under these conditions, gametocytes were visible from 7th day.

#### In vitro analysis

The drug plates were prepared in pmol/l as per the WHO in vitro microtest procedure by SMI assay [15]. CQ, AS (Sigma-Aldrich) stock solutions of 10 mM concentrations were diluted two folds to coat the drug plates [16]. A 50 μl of the blood media mixture (BMM) was added in the pre-dosed drug plate in triplicate with serial drug dilutions and then incubated in CO_2_ gas mixture at 37 °C for 24–30 h for schizont maturation. After 24 h incubation, a thin smear was prepared from the control well to observe mature schizonts and if more than 10% schizonts (with more than 3 nuclei) were seen in the control wells, it was considered to be valid. The thick smears were Giemsa-stained and the schizonts were counted out of a total of 200 asexual parasites for calculating IC_50_ values. For the test, the 3D7 was used as the reference strain with a mean 19.1 nM and 2.4 nM IC_50_ value for CQ and AS, respectively.

#### Detection of gametocyte genes

*Pfs 25* and *Pfg377*gene: The PCR analysis for *Pfs25* and *Pfg377* genes in the patient samples were carried out using the PCR conditions with high fidelity Taq DNA polymerase as described previously (ThermoFisher Taq) [16, 17].

#### Sequence analysis

The nested PCR products for region 3 of *Pfg377* were purified and sequenced using the cycle sequencing reaction with ABI Big Dye terminator version 3.1. The *Pfg377* gene of 3D7 strain (genID: Pf 3D7_1250100) was used as reference for sequence comparisons of the field isolates. The sequences were then assembled, aligned and edited using MEGA 6 and Finch TV program [13].

### Statistical analyses

The aligned *dhfr*, *dhps* and *Pfg377* gene sequences were used to determine the number of haplotypes. Also ɵ and π, the two measures of nucleotide diversities were estimated for each parasite population in each region [18]. The π nucleotide diversity parameter is based on the average number of pairwise nucleotide differences per site and ɵ estimates is dependent on the number of segregating sites. All these were estimated using DnaSP version 5.10.01 [18].

## Results

### Samples

Out of the total 143 samples analysed for mixed infections of *Plasmodium* spp. six samples were found to be of mixed infections with *P. falciparum* and *P. vivax*. Only *P. falciparum* mono-infections 122 sample (single parasite infections) were analysed in this study and *P. vivax* and co-infections were not further proceeded for any other examination. No follow up study was done to evaluate the parasite clearance in the patients hence the clinical data on delayed asexual stage parasite clearance was not available.

### Drug resistance genes

Table 1 gives the distribution of these genes in the three studied population.

**Table 1:** Percentage of drug resistance mutations seen in the parasite population at the three sites of the country viz Ranchi, Raipur and Mewat

Region	Drug resistance genes			
	*Pfcrt* (K76T)	*Pfmdr*-*1* (N86Y)	*Dhfr* (16, 51, 59, 108, 164)	*Dhps* (436, 437, 540, 581, 613)
Ranchi, Mutant	18 (81.81)	20 (90.9)	16 (72.7)	18 (81.81)
Raipur, Mutant	31 (77.5)	22 (55)	25 (62.5)	24 (60)
Mewat, Mutant	31 (51.7)	33 (55)	32 (53.3)	31 (51.7)

Values in the parenthesis indicate the percentage of isolates in the specific category

### *Pfcrt*, *Pfmdr*-*1* gene alleles and *k13*

*Pfcrt* gene was examined for K76T allele and was successfully amplified in 122 of field isolates. After digestion with ApoI enzyme the mutant allele 76T was seen in 80 (65%) in samples and 42 (37.4%) were wild type isolates indicating their sensitivity to CQ (Table 1).

The *Pfmdr*-*1* gene amplicons were digested by restriction enzyme AflIII. Upon digestion, different patterns of N86Y were observed where wild N-codon was in 47 isolates (38.5%) wild and mutant Y-codon in 75 isolates (61.4%) and mixed N/Y in two isolates (2.46%). We did not observe any significant correlation between the *Pfcrt* K76T and *Pfmdr*-*1* N86Y SNPs in the study (Table 1). The *k13* amplified did not reveal any synonymous or non-synonymous prevalent mutations in the field isolates.

### *dhfr* and *dhps* genes

The *dhfr* and *dhps* genes for SP were sequence analysed for the presence of mutations in 122 and 121 isolates respectively. Different isolates showed presence of different SNPs and a rise in double and triple mutations was observed in both the genes (Table 2). In *dhfr* gene the mutant 59R associated with high-level SP resistance was found to be 42.6% and for codons 51I, 108N and 164L mutants were observed at 31.9%, 17.2% and 7.3% in the population respectively. The wild genotype for *dhfr* was seen at higher frequency in Raipur with 37.5% followed by 27.2% in Ranchi and 22.9% in Mewat regions.

**Table 2:** Nucleotide diversity in *dhfr* and *dhps* allelic loci in sub-population areas with their different test parameters

No. of samples (n)	*dhfr*			*dhps*		
	Ranchi	Raipur	Mewat	Ranchi	Raipur	Mewat
	22	40	60	22	40	60
Mutant (%)	16 (72.70)	25 (62.50)	32 (53.30)	18 (81.80)	24 (60)	31(51.60)
SNP (n) total number of mutant alleles found in different regions	27	46	52	38	41	50
Nucleotide diversity: Ɵ	1.09	0.9	0.8	1.09	0.4	1.07
Pairwise difference: π	1.13	1.11	1.35	0.8	1.02	1.31
Test of neutrality						
Tajima’s D	0.094473	0.437752	1.276397	− 0.747549	2.225384	0.526865
Fu and Li’s D*	0.142505	− 0.07277	0.986851	− 0.81047	0.771237	1.080688
Fu and Li F*	0.137096	− 0.698455	1.197038	− 0.842395	1.297203	1.00836

Tajima’s D, Fu, Li’s D* and Li F* test used for standardized measure of average number of mutations between pairs in the sample. Nucleotide diversity (Ɵ) and pairwise difference (π) indicates the distribution of mutation rate which varies among the regions. # Total number of field isolates in the parenthesis for that particular region. P value for all the three sites for the two genes was found to be non-significant (< 0.05)

The *dhps* gene mutations at codon 436F was 47.4% and 540E was 42.6% respectively whereas 437G, 581G and 613T occured at 7.3%, 5.7% and 1.6%, respectively. In *dhps* gene the wild type field isolates were highest in Mewat region with 23.7% followed by 13.1% in Raipur and 3.2% least in Ranchi (Table 2). The triple mutation associated with high level SP resistance 51I R59 N108 was highest in Mewat followed by Raipur and Ranchi with 36%, 35.4% and 20.4%, respectively.

### Genetic diversity analysis

The sequence analysis of *dhfr* and *dhps* genes revealed highest nucleotide diversity in Ranchi for both the genes where the ɵ and π estimates of *P. falciparum* isolates showed an increase in the nucleotide diversity on comparison with Raipur and Mewat (Table 2). The π values suggest slight increase in intermediate frequency mutations in the population. The neutrality tests show no statistically significant results for the tests carried out for any divergence in both genes in the study population (Table 2).

### Sequence analysis

**Fig. 2:**
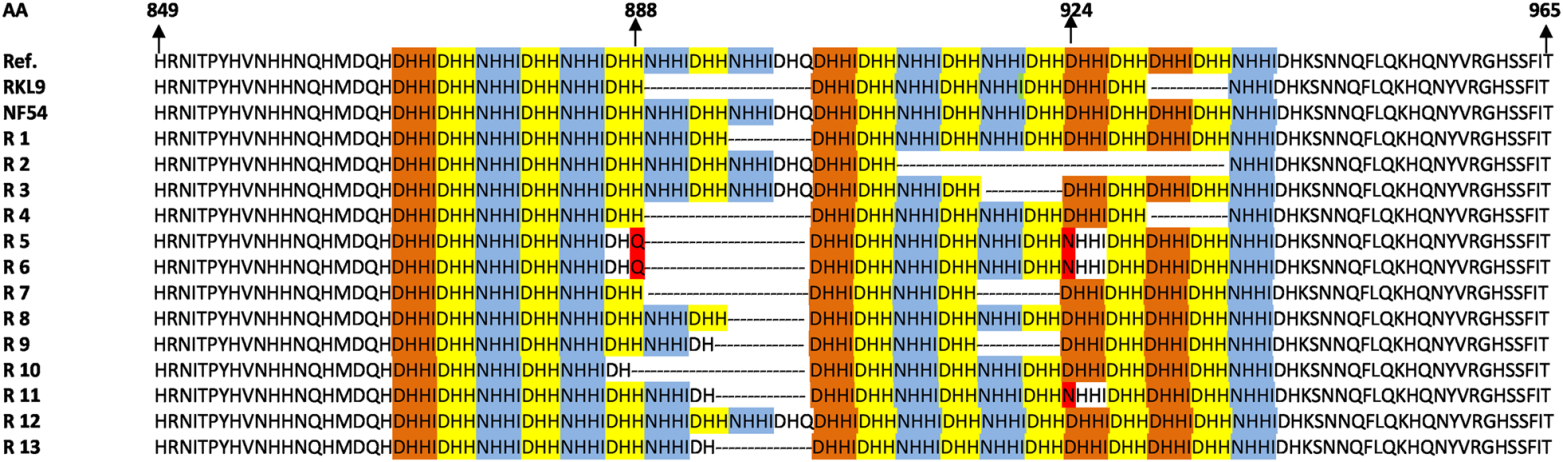
Sequence analysis of *Pfg377* alleles in the field isolates of *P. falciparum* with three reference strain 3D7, NF54 and resistant strain RKL9. The colour-coded base pair represent the repeated aminoacid (AA) sequence viz DHHI formed four times, DHH nine times and NHHI repeated seven times; varying within isolates. The dotted symbol shows various deletions-21 bp, 42 bp, 63 bp, 83 bp. Two new mutations in the sequence at codon position 888 histidine (H) to glutamine (Q) (in turquoise blue colour) and at 924 position aspartic acid (D) to asparagine (N) (in green colour) are also reported

The region 3 of *Pfg377* gene was included in the study as it is the most polymorphic region and hence used in allele typing. Sequencing the 3 regions of the *Pfg377* gene was carried out for 122 isolates revealed the deletion of 21 bp and multiples of 21 bp. On comparison with NF54 reference sequence, the isolates showed seven different types of deletions viz NHHIDHQ, HNHHIDH, HNHHIDHHNHHIDH, DHHIDHH, NHHIDHH, NHHIDHHNHHIDHQ and NHHIDHHNHHIDHHDHHIDHHDHHIDHH amino acid fragments. The most commonly occurring deletion was HNHHIDH, found in 42 isolates. The isolates with no deletions exhibited DHHI, DHH and NHHI sequence repeats 4, 9 and 7 times, respectively. These repeats occurred at different frequencies in the other forty isolates repeating these sequences 4, 8 and 6 times, respectively. On the basis of these deletions five types of genotype was present in the field isolates and He for *Pfg377* gene was found to be 0.75. Two new mutations-D924N and H888Q mutation was found in four and nine isolates respectively on sequence analysis (Fig. 2).

Combining the mutational analysis of the *dhfr*, *dhps* and *Pfg377* genes 18 haplotypes were observed in the present study in all the populations (Table 3). Among these haplotypes most prevalent was A10 commonly seen in Raipur and haplotype A18 depicted the wild population (n = 44) seen distributed randomly in Mewat (45%), Raipur (37.5%) and Ranchi (9%). The combined results of *dhfr* and *dhps* mutational analysis reveals highest polymorphisms in parasite populations from 77.7 and 81.8% respectively in Ranchi.

**Table 3:** The distribution of haplotypes (A1–A18) based upon the mutation codon differentiation for *Pfcrt*, *Pfmdr*-*1*, *Pfdhfr*, *Pfdhps* and *Pfg377* genes

Haplotype	*Pfcrt*	*Pfmdr*-*1*	*dhfr*	*dhps*	*Pfg377* (bp *deletions*)	No. of isolates	% frequency
A1	76T	86Y	51I, 59R, 108N, 164L	436F, 437A, 540E, 581A, 613T	21, 63	1	0.8
A2	76T	86Y	51I, 59R, 108N, 164I	436F,437G, 540E, 581A, 613A	21, 63	1	0.8
A3	76T	86Y	51I, 59R, 108N, 164I	436F, 437A, 540E, 581A, 613A	21, 63	1	0.8
A4	76T	86Y	51I, 59R, 108N, 164I	436F, 437A, 540E, 581A, 613A	21	7	5.7
A5	76T	86Y	51N, 59R, 108S, 164I	436F, 437A, 540E, 581A, 613A	63	9	7.3
A6	76T	86Y	51N, 59C, 108S, 164I	436F, 537A, 540E, 581A, 613A	21, 63	1	0.8
A7	76T	86Y	51I, 59C, 108S, 164I	436F, 437A, 540K, 581A, 613A	83	1	0.8
A8	76T	86Y	51N, 59C, 108S, 164L	436S, 437A, 540K, 581A, 613A	63, 42, 83	1	0.8
A9	76T	86Y	51N, 59C, 108N, 164I	436S, 437A, 540K, 581A, 613A	21, 63, 42	2	1.6
A10	76T	86Y	51N, 59R, 108N, 164I	436F, 437A, 540K, 581E, 613A	21	21	17.2
A11	76T	86Y	51I, 59R, 108N, 164L	436F, 437G, 540E, 581A, 613A	21	8	6.5
A12	76T	86Y	51I, 59R, 108N, 164I	436F, 437A, 540E, 581G, 613A	21	5	4
A13	76T	86Y	51I, 59R, 108N, 164I	436F, 437A, 540K, 581G, 613T	21	2	1.6
A14	76T	86Y	51I, 59C, 108N, 164I	436F, 437A, 540K, 581A, 613A	–	1	0.8
A15	76T	86Y	51N, 59C, 108S, 164I	436F, 437A, 540K, 581A, 613A	21	3	2.4
A16	76T	86Y	51N, 59C, 108S, 164I	436S, 437A, 540K, 581A, 613A	–	12	9.8
A17	76K	86Y	51N, 59C, 108S, 164I	436S, 437A, 540K, 581A, 613A	–	2	1.6
A18	76K	86N	51N, 59C, 108S, 164I	436S, 437A, 540K, 581A, 613A	–	44	36

A18 is the wild type haplotype (drug resistance genes) found in the studied isolates

### Phylogenetic analysis of *dhfr*, *dhps* and *Pfg377* genes

**Fig. 3:**
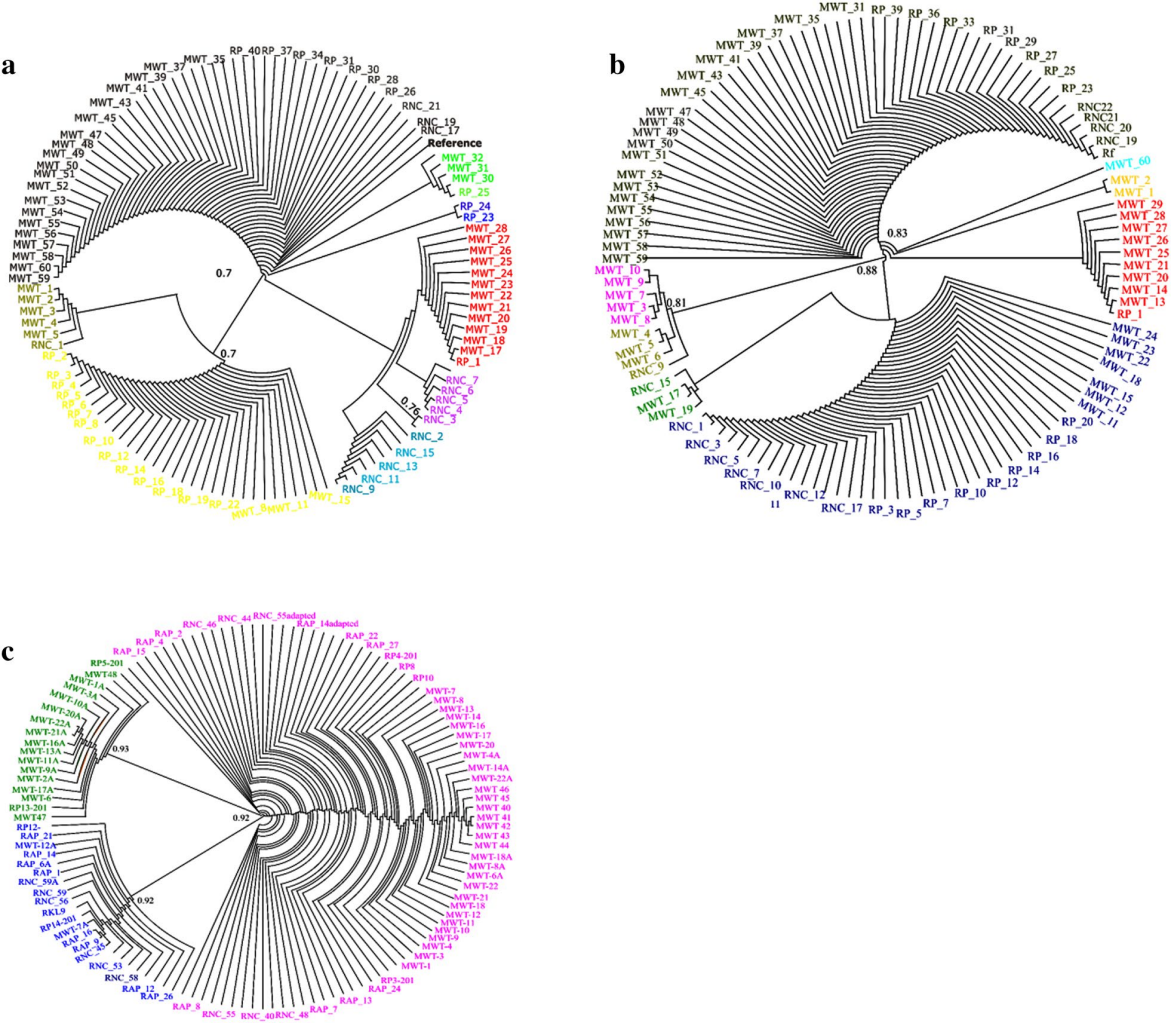
**a** Phylogenetic analysis of *dhfr* genotypes in individual isolates determined by radial cladogram. The different colour in the cladogram depicts different mutations seen in the field isolates. **b** Phylogenetic analysis of *dhps* genotypes in individual isolates determined by radial cladogram. **c** Phylogenetic analysis of *Pfg377*genotypes in individual isolates determined by radial cladogram. The alignment of the field isolates is based on their sequence variations

To identify the existing genetic diversity in the parasite population the radial cladogram network was constructed between the drug resistance loci *dhfr*, *dhps* and gametocyte gene *Pfg377* by using Dendroscope version 3.5.9 software [19]. The genetic relatedness is determined by clade in each phylogeny tree which signify the similarity in the various isolates. In *dhfr* phylogeny tree we find that the isolates were divided in two clades: the first clade was formed by the wild type isolates along with the reference strain and the second clade comprises of isolates having mutant alleles. The mutant clade consists of different sub-group clades within isolates having single, double or triple mutations (Fig. 3a). The sub-group clade subdivisions in different colours depicts the different mutations in the populations. The most predominant mutation clade in the cladogram consists of 59 and 108 codons while the least prevalent 108 mutations made the other clade. The same sequence relatedness was also found in *dhps* allelic profiling (Fig. 3b). The most predominant in this allelic profile was 436 followed by 540 codons and the least one was codon 613 position. In *Pfg377* the clades are similar with variations due to short sequence deletions and repeats among the population (Fig. 3c). The gene distribution shows no particular pattern for any mutant alleles of *dhps* and *dhfr* distributed across the country and to any particular region (Fig. 3a, b). Similarly, for *Pfg377* gene too no distinct pattern for deletions or repeats were seen, with all randomly distributed in the population (Fig. 3c).

### Culture studies

The cryopreserved samples were subjected to in vitro culture for revival and 44 isolates adapted in culture. After successful establishment of culture adapted samples, the isolates were put for gametocyte production in vitro. After 15 days of culturing in laboratory, 10 field isolates produced gametocytes and various stages I–V were identified by microscopy. From 7th day onwards post sub-culture, different stages of gametocytes were seen in smears. In two isolates no gametocyte production was seen though the asexual stages were identified in smears during culture adaptation in laboratory.

### In vitro drug sensitivity assay

**Fig. 4:**
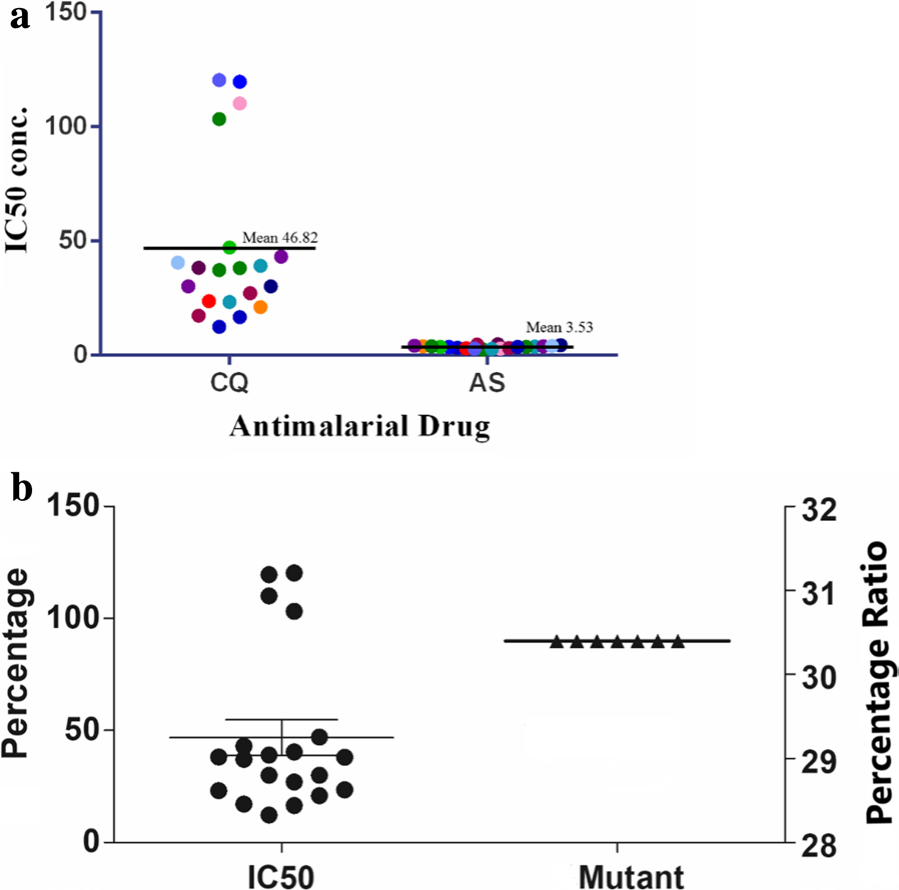
**a** The IC_50_ pattern of the field isolates (represented by coloured dots) tested for chloroquine (CQ) and artesunate (AS) drug. **b** Graph showing the comparative analysis of inhibitory concentration (IC_50_) of adapted isolates with K76T (*Pfcrt*) mutant isolates after sequencing analysis (X-axis). The percentage values of field isolates for both analysis shows the accordance of results in them

Due to limited volume of cryopreserved samples it was technically difficult to analyse with all anti-malarial drugs hence only primary drugs-CQ and AS in vitro sensitivity assay was performed using the SMI assay. The CQ resistant isolates showed schizont maturation at 8 pmol or more and for AS no resistance was seen in isolates. The inhibitory concentrations (IC_50_) for the CQ and AS, drugs were found in the range of 10.11–113.2 nM, 2.26–4.08 nM, respectively in the evaluated field isolates (Fig. 4a). The IC_50_ values for CQ have shown a remarkable reduction on comparison with the previous available data, whereas a slight increase in the IC_50_ values for AS were observed. On comparing the IC_50_ values with the sequencing data of the field isolates it was observed that the isolates which had high IC_50_ values also harboured the K76T mutations in the *Pfcrt* gene (Fig. 4b). Two isolates which were resistant to CQ (MWT 16 and 17) also had high IC_50_ for AS values indicating an increase in the tolerance value for AS drug. MWT-14 and MWT-20 were found to have high IC_50_ values for both the tested drugs though MWT-14 was a single clone and MWT-20 a multiclone on analysis. Due to the unavailability of required sample volume the Ring-stage Survival Assay (RSA) for the samples was not carried out and only CQ and AS IC_50_ values were analysed in limited samples.

## Discussion

The malaria situation continues to prevail even after intensive control efforts and remains persistent in India; as India is a major hot-spot for malaria transmission due to its unique favourable topography with respect to malaria survival [20]. Drug resistance is a major hurdle in malaria control not only in the country but worldwide too [21]. Continuous monitoring of the drug resistance provides warning signals to drug policy makers. The emergence of drug-resistant parasites in India is still on the rise even after the introduction of ACT in the country [4]. Genotyping of *Pfcrt*, *Pfmdr*-*1*, *dhfr, dhps* genes and *k13* was carried out in field isolates of *P. falciparum* collected from Ranchi, Raipur and Mewat to determine the prevalence of polymorphisms in these genes along with the neutral gametocyte gene viz *Pfg377*. Not many such studies have been reported from India.

In the study, non-drug resistant *Pfg377*gene showed lot of genetic diversity in the field isolates which agrees with the observed random association between the loci and absence of regional differentiation among the *P. falciparum* isolates is in accordance with other reports [22, 23]. CQ resistance (CQR) was monitored on the point mutation detected in the *Pfcrt* and *Pfmdr*-*1* genes by PCR–RFLP method where a high prevalence of mutant K76T *Pfcrt* and N86Y *Pfmdr*-*1* alleles was observed. This holds true with the previous studies stating the role of *Pfcrt* gene crucial in detection of CQR in *P. falciparum* field isolates [24–26]. No linkage disequilibrium (LD) between the N86Y and K76T mutations in *Pfmdr*-*1* and *Pfcrt* genes was observed and more number of samples are to be studied to compare the LD association in *P. falciparum* isolates. It is extremely important to routinely detect/analyse the pattern of drug resistance in malarial parasites which play not only an important role in the epidemiological surveys but also help in improvising/updating the anti-malarial drug policies of the country. The CQ resistance if *P. falciparum* is mainly linked to the mutation in K76T codon of *Pfcrt* gene followed by *Pfmdr*-*1* gene N86Y [24, 25].

SP are the partner drugs in the current ACT therapy of the country and we observed a high prevalence of more than one mutations in the *dhfr*, *dhps* genes of *P. falciparum* [27]. The double mutants 51I 108N in the *dhfr* gene were present at a higher frequency in the population as compared to the triple mutant 51I 59R 108N associated with high resistance for SP was only seen in the field isolates from Mewat [28]. It has been reported from Africa and South East Asia earlier that I51 R59 N108 are highly SP resistant haplotype and the presence of this genotype in the studied population reveals that the parasite population is possibly moving towards SP resistance where evidence of selection is seen earlier in *dhfr* gene than *dhps* gene [29, 30]. This indicates the possibility of high resistant genotypes evolving in the parasite population due to constant drug pressure experienced by the parasite [31]. The π values of nucleotide diversity were higher for *dhfr* and *dhps* genes in Mewat and similar in Ranchi and Raipur the endemic regions for malaria similarly documented from other places [32]. Analysing the SNPs in these drug resistance genes to find new mutations will help us to understand genetic changes leading to altered phenotypic traits better and also give an insight into the evolving genetic recombinations with respect to the current drug therapy [33]. The usage of ACT in the national drug policy since a decade is expected to decrease the CQ resistance and restore the CQ sensitive parasite population as such a change has been reported from several countries and 31.8% wild type of isolates in the parasite population was also seen in the present study [34].

Similarly, though the majority of isolates were found to be resistant to CQ, the IC_50_ observed value for CQ was comparatively low as seen in other reports [35]. This low value is indicative of the fact that the absence of CQ in malaria treatment has released the drug pressure with a possibility of moving towards being CQ sensitive in future as reported from other parts of the world [36]. The field isolates were found to be AS sensitive through with high IC_50_ values. Due to constant exposure to AS the increase in the drug pressure is likely to be responsible for increase in the IC_50_ values and another plausible explanation also could be the indiscriminate use of the drug resulting in slow rise of low level resistance in the parasite population [37]. In vitro drug sensitivity remains an important tool to assess the efficacy of the anti-malarial drugs. The change of anti-malarial treatment in the country from CQ to ACT is expected to decrease the CQR population and might be leading to restore the sensitivity to CQ as reported from other places [38]. The levels of parasitaemia varied in the infections and only 44 samples could be culture adapted. The difference in the growth pattern in different field isolates may be due to their difference in commitment to differentiate into asexual or sexual stages [39]. The expression profile of *Pfg377* gene was correlated with the efficiency of gametocyte production in the above isolates. It was observed that isolates which produce mature gametocytes in vitro also showed an increase in the*Pfg377* gene expression from 1.00 to 4.56-fold when compared to NF54 reference strain. The results suggested that a correlation between *Pfg377* gene expression and the ability to produce gametocyte (mature) by the isolate exists as also reported by others [40].

The allele typing of *Pfg377*-the sexual stage specific gene in the field isolates revealed several allele types present in them. By conventional PCR only the predominant genotype was detected in the PCR results and presence of more than one clone in a single infection were observed on sequence analysis. This shows that this gene can be effectively used for detecting the presence of gametocyte producing multiclones in the field isolates. The presence of different genotypes/clones in the same infection is an important source of transmission [8]. The large number of repeats found in this gene is responsible for the high variability seen in parasites thus allowing several parasite lines to merge and survive simultaneously leading to transmission dynamics [41]. It is understood that presence of mixed gametocyte genotypes in the same sample is responsible for harbouring gametocytes for a significantly longer period of time on comparison with a single genotype where it was cleared three times faster than multiple clones [42]. Natural infections of *P. falciparum* seem to harbour multiple genotypes in the same host which could be due to the transmission burden in endemic regions but still, this knowledge does not improve the understanding of existing multiplicity in parasite biology [43].

By substantiating the results obtained from the genome sequence of *P. falciparum* and phylogenetic analysis, a basic idea about the population genetic structure was inferred. The phylogenetic trees of all three genes divided the isolates into two clear clades that can potentially be divided into further subgroups. However, no obvious cluster was observed and the distribution of isolates within the subgroups was random. There was no apparent trend for sequences of the same geographic region to be more closely related. The non-availability of wild-type of isolates limits our understanding of wild type parasites *vs* drug-resistant parasites as most of the infections had at least single/double SNPs in drug resistance genes with extensive diversity present in the *Pfg377* gene. More comprehensive studies like this are needed to understand the emerging complexity in the parasite population. The gametocyte data in relation to diversity level is too limited in Indian context. Our main focus to include this data was to generate data for any type of correlation between the mutation in drug resistance genes and gametocyte gene as these gametocyte genes play a crucial role in transmission as it gives baseline data for further work in this area.

## Conclusion

This study provides information on drug resistance phenotypes, which have a critical role to play in malaria transmission. The increasing mutation rates in *dhfr*, *dhps* genes, existing diversity in the *Pfcrt*, *Pfmdr*-*1* and *Pfg377* genes, increase in the IC_50_ values for AS, in the parasite population indicates that as the parasite genetic diversity is multifactorial; the emergence of drug resistant genotypes need to be tackled by multipronged approach. The knowledge of the host dynamics with its epidemiological and evolutionary consequences is important for intervention measures to be successful and efforts should also be made to prevent transmission of *P. falciparum*. To assess the resistance dynamics and the surveillance of molecular markers of drug resistance is essential as it helps in selecting the most effective treatment and also should help in malaria intervention programmes.

## Data Availability

The data used in this study is available from the corresponding author upon reasonable request.

## Abbreviations

*Pf*: *Plasmodium falciparum*
*crt*: chloroquine resistance transporter
*mdr*: multidrug resistance
*dhfr*: dihydrofolate reductase
*dhps*: dihydropteroate synthase
*msp*: merozoite surface protein
IC_50_: inhibitory concentrations
SMI: schizont maturation inhibition
MOI: multiplicity of infection
BMM: blood media mixture
CQ: chloroquine
AS: artesunate
ACT: artemisinin-based combination therapy
RFLP: restriction fragment length polymorphism
